# Systematic Scale-Up of Mechanochemical Paracetamol–Oxalic Acid Cocrystal Synthesis across Multiple Milling Technologies

**DOI:** 10.1021/acs.oprd.6c00052

**Published:** 2026-05-13

**Authors:** Jan-Hendrik Schöbel, Dhyanesh Gopinath, Michael Felderhoff

**Affiliations:** Department of Heterogeneous Catalysis, 28314 Max-Planck-Institut für Kohlenforschung, Kaiser-Wilhelm-Platz 1, Mülheim an der Ruhr D-45470, Germany

**Keywords:** mechanochemistry, scale-up, cocrystals, attritor mill, drum mill, planetary ball mill

## Abstract

Mechanochemical cocrystallization provides an efficient, solvent-minimized route for the production of pharmaceutical cocrystals. Although the small-scale synthesis of cocrystals is well established, strategies for the gradual scale-up to multigram and kilogram quantities remain limited. By systematically analyzing and comparing different scale-up stages, this study investigates the scale-up of paracetamol-oxalic acid (PCA-OXA) cocrystals using a range of milling technologies, including planetary ball mills, attritor mill, and drum mill. We aim to provide practical guidance on the capabilities and limitations of these devices and identify suitable milling parameters and strategies that ensure complete conversion and high product quality as batch sizes increase. In practice, planetary ball mills were used to prepare multigram quantities, while attritor mills generated hundred-gram batches under dry milling conditions. At the kilogram scale, drum milling required liquid-assisted grinding (LAG) with ethyl acetate to prevent caking and achieve full conversion. The results show that the gradual scale-up of PCA-OXA cocrystals is feasible when milling conditions and appropriate milling technologies are carefully selected, maintaining high yields and product quality across different scales. The findings contribute to a better understanding of the opportunities and limitations of mechanochemistry in pharmaceutical cocrystal development and offer guidance for the transition from laboratory-scale discovery to larger-scale production.

## Graphical abstract

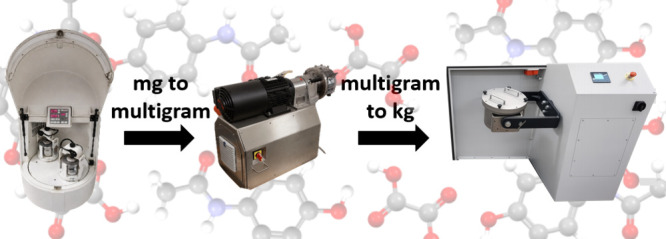

## Introduction

Pharmaceutical cocrystals are a valuable strategy to overcome limitations in the physicochemical and mechanical properties of active pharmaceutical ingredients (APIs).^1^ By forming a single crystalline phase that incorporates an API and a pharmaceutically acceptable coformer through noncovalent interactions, cocrystals can enhance attributes such as solubility, stability, compressibility, and bioavailability without altering the molecular structure of the drug itself.^2^ This makes them particularly attractive in the development of solid oral dosage forms, where poor water solubility and poor mechanical properties often hinder formulation success.

Among the many APIs studied for cocrystal formation, paracetamol (PCA) stands out as one of the most widely used analgesic and antipyretic drugs globally and is included on the World Health Organization’s (WHO) list of essential medicines due to its safety and accessibility.^3^ However, despite its widespread use, PCA suffers from notable drawbacks such as low aqueous solubility, poor tabletability, and relatively weak analgesic effects, which often necessitate high dosing. These limitations have driven significant interest in the development of paracetamol-based cocrystals as a means of improving its functional performance.^4^

Pharmaceutical cocrystals are commonly prepared using techniques such as solution crystallization, slurry methods, and solid-state grinding.^1c,5^ For paracetamol, several coformers, including oxalic acid (OXA),^4a,4c–4e^ citric acid (CIT),^6^ theophylline (THP),^4a^ phenazine (PHE),^4a^ and trimethylglycine (TMG),^7^ have been successfully paired with paracetamol to yield cocrystals that demonstrate improved physicochemical and mechanical properties. For instance, the paracetamol-oxalic acid cocrystal forms a dense hydrogen-bonded network that enhances both mechanical strength and compactability, making it a suitable model system for formulation studies.

Mechanochemistry,^8^ in which chemical transformations are induced by mechanical force rather than by heat, light, or electricity, offers a green alternative to solution-based methods, aligning with broader sustainability goals in pharmaceutical manufacturing.^9^ In particular, mechanochemical methods such as neat grinding and liquid-assisted grinding (LAG) have proven effective for the synthesis of cocrystals without the need for bulk solvent or extensive purification.^10^ Moreover, these methods often enable faster crystallization and are compatible with a wide range of API-coformer combinations. Despite these advantages, a critical barrier to industrial adoption of mechanochemical cocrystallization is the lack of understanding around process scalability. While small-scale synthesis in laboratory with planetary ball mills or vibratory mills is well established, few studies have systematically addressed the scaling up of these processes to the multigram or kilogram scale.^11^ Challenges such as heat management, energy transfer, mixing efficiency, and homogeneity become more pronounced at larger scales and differ depending on the milling technology used. In our previous work, we have demonstrated the feasibility of cocrystal formation on multigram to kilogram scales using various mechanochemical devices. Specifically, we reported the successful synthesis of pharmaceutical cocrystals in an eccentric vibratory mill,^12^ an attritor mill,^13^ and a drum mill,^14^ highlighting their potential for larger-scale applications. These studies, primarily conducted on ibuprofen-based cocrystals, confirmed that cocrystal formation is not limited to small-scale laboratory settings and can be achieved with high yield and purity under suitable conditions.

Different milling devices induce distinct types of mechanical energy, which influence particle size reduction, mixing efficiency, and potential material wear. Planetary ball mills combine the rotation of the milling jars with the revolution of the milling platform, generating high-energy impacts and shear forces that enable rapid particle comminution and cocrystal formation at milligram to gram scales. Attritor mills, particularly high-energy variants, consist of a vertical or horizontal shaft with attached blades that vigorously agitate the milling media at high rotational speeds, producing frequent collisions and intense impact forces. This configuration allows efficient energy transfer and scalability to larger batches. In contrast, drum mills operate primarily via gravity-driven cascading or cataracting motion, resulting in lower impact energies and gentler mixing, which reduces abrasion compared to other mill types.

The focus of our earlier investigations was primarily on proving the capability of these milling devices to produce cocrystals at scale, rather than systematically analyzing the scale-up process itself. Building on these results, the present study shifts focus to the gradual scale-up process itself, using paracetamol cocrystals as a model system. Paracetamol was chosen because, despite being well studied in small-scale mechanochemical experiments, its cocrystals have not yet been explored at larger scales. Since mechanochemistry currently lacks standardized parameters for translating processes across different devices, scaling up remains particularly challenging. By following the synthesis from milligram to kilogram quantities across different milling technologies, we aim to identify practical challenges, machine-specific effects, and key parameters influencing scale-up. Successful scale-up is assessed in terms of high conversion to the desired cocrystal phase, phase purity, and reproducibility across repeated experiments, ensuring reliable and consistent results at each scale.

## Results and Discussion

### Screening of Suitable Coformers

Initially, a selection of coformers reported in the literature was evaluated for their ability to form cocrystals with PCA. The screening focused on compounds with properties favorable for subsequent scale-up, including low cost, ready availability, nontoxicity, reproducibility, and suitable crystallization behavior. The coformers selected for testing were oxalic acid (OXA), citric acid (CIT), theophylline (THP), phenazine (PHE), and trimethylglycin (TMG).

Co-crystallization reactions were conducted in a planetary ball mill, where PCA and each coformer were milled together until complete conversion was achieved. The resulting mixtures were analyzed by differential scanning calorimetry (DSC) and powder X-ray diffraction (PXRD) to identify the characteristic melting behavior and reflections of the desired cocrystals (Figure 1).

**Figure 1:**
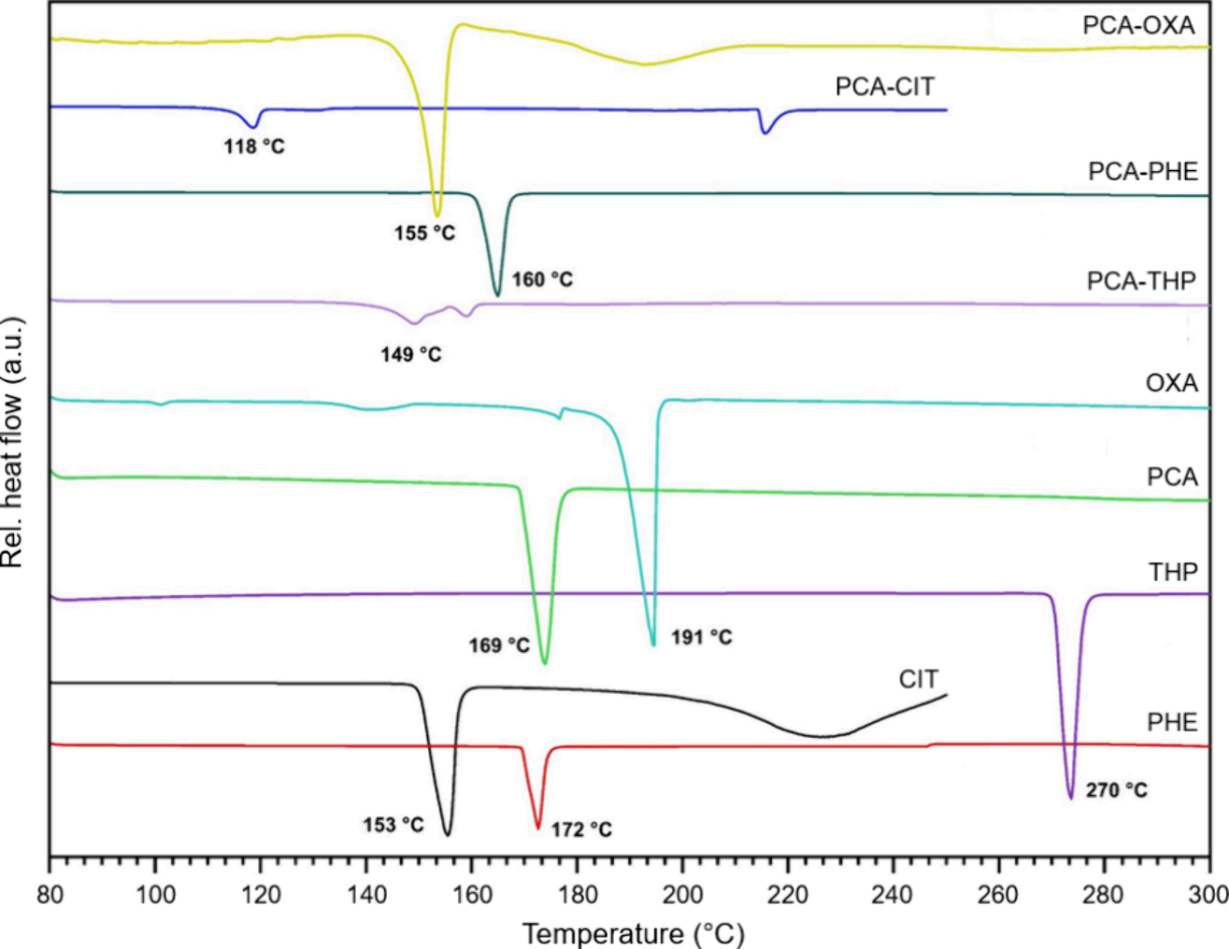
DSC thermograms of PCA, selected coformers, and the corresponding PCA cocrystals. Thermograms are shown from top to bottom for PCA-OXA (yellow), PCA-CIT (blue), PCA–PHE (petrol), PCA-THP (light purple), OXA (cyan), PCA (green), THP (violet), CIT (black), and PHE (red). Distinct thermal events and melting transitions observed for the cocrystals compared to the parent compounds indicate the formation of new solid phases.

Among the tested coformers, TMG did not yield the PCA-TMG cocrystal. The reaction formed a wet, poorly crystalline compound that was unsuitable for DSC or PXRD analysis. With CIT as coformer, the expected melting behavior of PCA-CIT (two endotherms at 72.1 and 94.1 °C) was not observed in the DSC thermogram.^6b^ Testing of alternative reaction conditions under neat milling (NM) or liquid-assisted grinding (LAG) conditions did also not yield the desired cocrystal (see ESI, Figures S3 and S4). THP (mp 270 °C) exhibited only minimal conversion within a reasonable milling time and was therefore excluded from further investigations.^4a^ In contrast, milling PCA with OXA or PHE successfully yielded the corresponding cocrystals PCA-OXA (mp 155 °C) and PCA–PHE (mp 160 °C),^4a^ whose formation was further confirmed by PXRD (see ESI, Figure S5). Considering that the PCA-OXA system met the criteria for larger-scale processing and has been previously reported to enhance both mechanical strength and compactability in tablet formulations, it was selected as the model system for subsequent scale-up studies.^4a,4c–4e^

### Scale-Up to Gram and Multigram Quantities Using Planetary Ball Mills

Following the screening of potential coformers, the effect of increasing reaction scale was investigated through the mechanochemical synthesis of PCA-OXA in a Fritsch Pulverisette 7 planetary ball mill (P7), covering scale-up at the lower-gram level. The machine′s working principle is based on the planetary motion: the bowls, loaded with sample and grinding media, rotate around their own axes while simultaneously revolving around a central main disk in the opposite direction, resulting in strong impact and friction forces. This device is equipped with two working stations, allowing the simultaneous synthesis of two batches using milling jars with a maximum capacity of 45 mL.^15^

For an initial experiment, 500 mg of PCA and 298 mg of OXA (1:1 molar ratio) were milled at 650 rpm in a 12 mL stainless steel jar, containing 50 × 5 mm stainless steel milling balls (BPR = 26.9). Analysis of the resulting mixture by PXRD confirmed substantial cocrystal formation after 35 min of milling, as evidenced by the PXRD pattern showing a strong characteristic reflection at 2θ = 27.8° (Figure 2). However, conversion was not yet complete, as indicated by the remaining paracetamol reflections at 2θ = 12.1° and 2θ = 13.9°. Milling was therefore continued, with periodic sampling and analysis, until complete cocrystallization was achieved after 4 h. These milling parameters were subsequently applied to a larger 45 mL milling vessel, while maintaining the same reactant masses. Despite the increased vessel volume, the BPR remained constant at 26.9, and complete cocrystallization was again achieved after 4 h, indicating that scaling up the vessel while maintaining the BPR preserves the energy input per unit mass and thus the reaction kinetics. When the total reaction mass was increased 5-fold, to 2.5 g of PCA and 1.5 g of OXA, complete cocrystallization was achieved after 3 h of milling. Under these conditions, the effective BPR decreased to 5.4 for 50 × 5 mm balls and to 2.7, when 25 × 5 mm balls were used. Despite the substantially lower BPR, no prolongation of reaction time was observed, indicating that the cocrystallization process is not strongly limited by energy input once a critical threshold is exceeded. This suggests that, under the applied milling conditions, the reaction kinetics are governed by solid-state transformation processes rather than by BPR alone. As approximately 4 g represented the upper limit for efficient mixing in the 45 mL vessel of the P7 mill, larger milling vessels and mills capable of handling higher volumes were subsequently tested.

**Figure 2:**
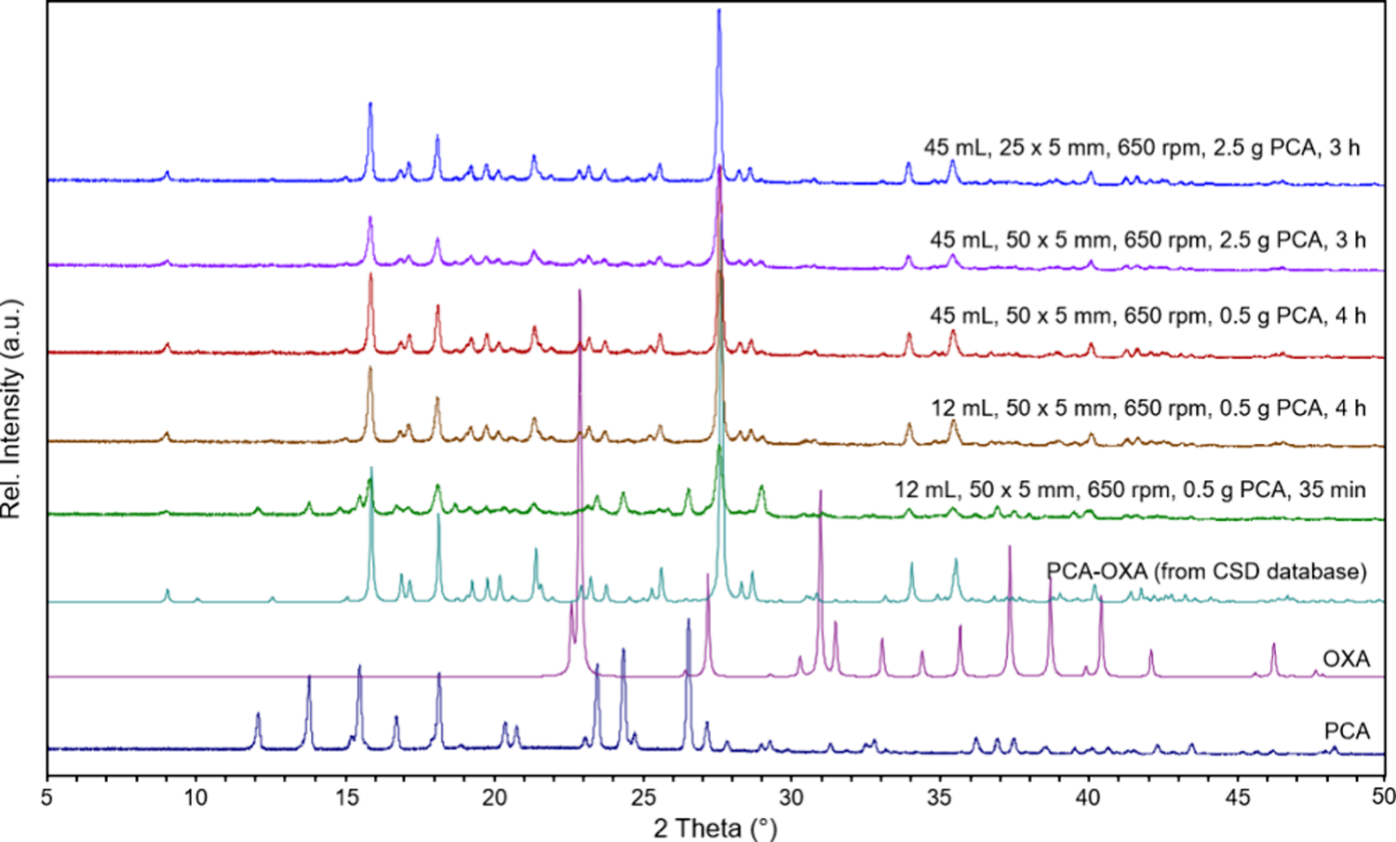
PXRD patterns of PCA-OXA synthesized in a P7 planetary ball mill under various conditions. Experimental parameters (jar size, number and diameter of milling balls, rpm, scale, and reaction time) are indicated above each pattern. Representative reflections are observed at 2θ = 9.0°, 10.0°, and 27.8° for PCA-OXA, at 12.1° and 13.9° for PCA, and at 31.0° for OXA. Pure PCA was measured as a reference and OXA (CSD 929767) and the PCA-OXA cocrystal (CSD 720368) from the Cambridge Structural Database (CSD) was included for comparison.

To further investigate the transition to larger scales, experiments were conducted using the Fritsch Pulverisette 5 planetary ball mill (P5). This device operates on the same planetary principle as the P7, but is designed for larger sample volumes and can accommodate grinding bowls of up to 500 mL in four working stations. The initial reaction was carried out using 5 g of PCA and 3 g of OXA (Figure 3). The P5 experiment employed larger milling balls (18 mm diameter compared to 5 mm in the P7) and fewer balls (12 vs 50). Additionally, the milling frequency was reduced from 650 to 250 rpm. This reduction was necessary due to the larger vessel size and mass, which limit the maximum achievable rotational speed and ensure safe operation of the machine, preventing excessive heating or mechanical stress. Despite these changes, the energy transfer in this experiment was sufficient to achieve complete cocrystallization, presumably due to the higher energy per impact delivered by the larger milling balls. Full conversion was achieved within 4 h of milling.

**Figure 3:**
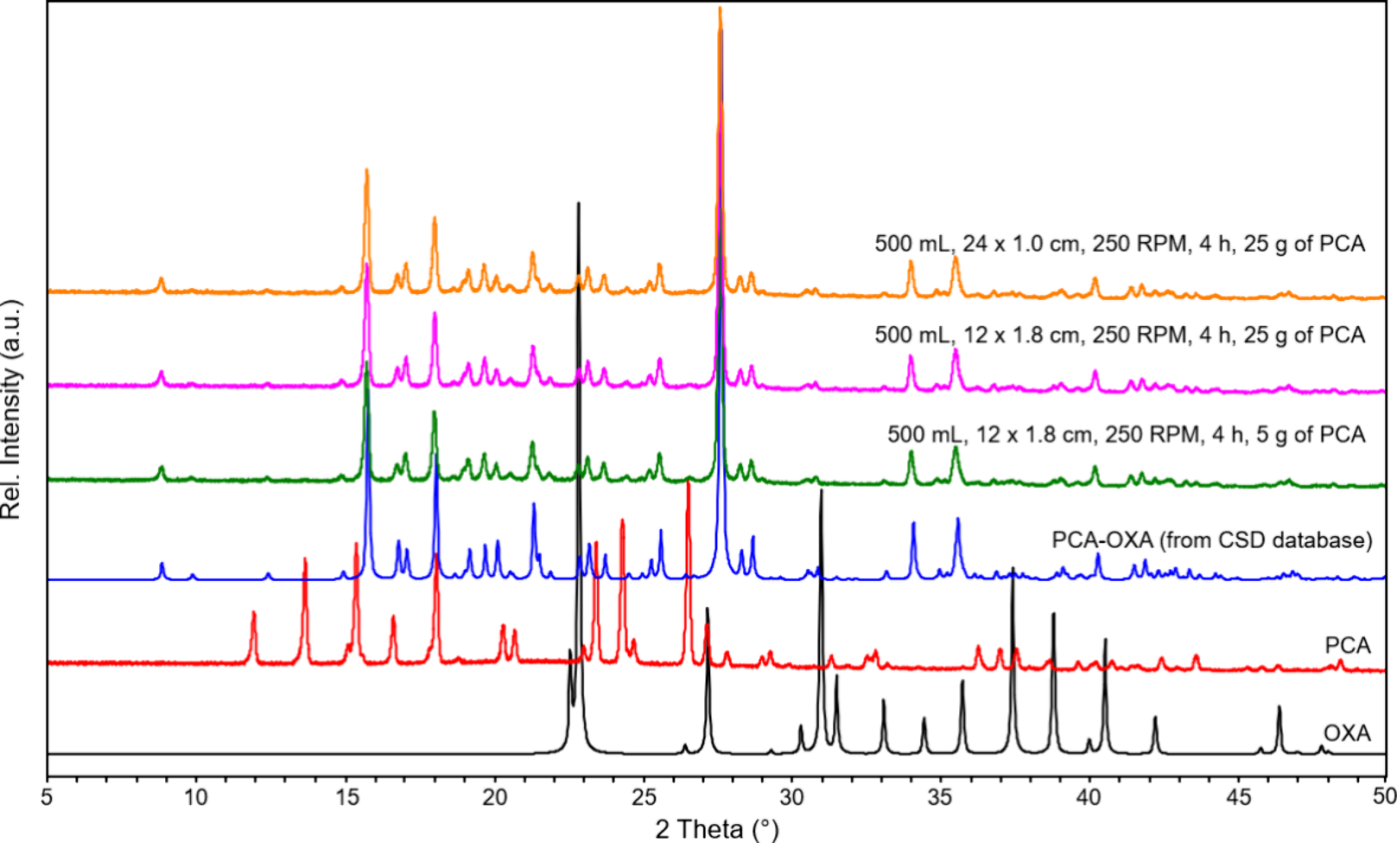
PXRD patterns of PCA-OXA synthesized in a P5 planetary mill under various conditions. Experimental parameters (jar size, number and diameter of milling balls, rpm, scale, and reaction time) are indicated above each graph. Pure PCA (red) was measured as a reference. OXA (black, CSD 929767) and the PCA-OXA cocrystal (blue, CSD 720368) from the Cambridge Structural Database (CSD) was included for comparison.

The reaction mass was then increased 5-fold, to 25 g of PCA and 15 g of OXA. Under these conditions, complete cocrystallization was again obtained after 4 h of milling (Figure 3). To assess the effect of ball size and number on the conversion rate, an additional experiment was conducted in the P5 using smaller balls (10 mm diameter) with a total of 24 balls. Interestingly, the conversion time remained the same as in the previous experiment, indicating that within this range of milling parameters, neither ball size nor number had a significant impact on the cocrystallization rate. Consequently, the ball-to-powder ratio (BPR) could be reduced in this set of experiments from very high values (BPR > 26 for 500 mg scales with 50 × 5 mm balls and 5 g scales with 12 × 18 mm balls) to lower values (BPR = 9.5 for 25 g scales) without compromising reaction efficiency. As expected, milling in planetary ball mills led to an increase in the bulk temperature inside the milling vessel due to the high friction generated by the characteristic planetary motion. Upon opening the jars after milling, temperatures in the range of 40–50 °C were typically observed, as measured using an IR thermometer. Experiments conducted at higher BPR values tended to reach the upper end of this range (e.g., ∼ 50 °C for BPR = 26 at the 500 mg scale), whereas lower BPR values resulted in temperatures closer to 40 °C.

### Scale-Up to Hundred-Gram Quantities Using an Attritor Mill

To extend the mechanochemical cocrystallization of PCA and OXA to larger scales, an attritor mill was investigated next. While the planetary mills demonstrated efficient cocrystal formation at smaller scales from mg to tens of grams, their scalability is inherently limited by the complex movement pattern of the milling vessel inside the machine, which makes industrial-scale application challenging. In contrast, attritor mills such as the Simoloyer, with reactor sizes available from 1 L up to 900 L, are industrially available and allow for processing larger quantities. The Simoloyer is a high-kinetic horizontal rotary ball mill equipped with a rotor shaft and attached blades that rotate at speeds of up to 1800 rpm, generating intense impact and collision forces.^13,16^

Initial experiments were carried out by milling 100 g of PCA and 60 g of OXA with 2 kg of stainless steel balls (d = 5 mm, BPR = 12.5) in a 1 L stainless steel reactor at 1000 rpm using the Simoloyer CM01 model from Zoz. As the reactor tends to heat up rapidly due to friction between the grinding media and reactor walls at high milling speeds, the process temperature was maintained at 18 °C by means of a cooling jacket surrounding the milling vessel (model: W01–1 lm). In contrast to experiments conducted in planetary ball mills, no measurable temperature increase was observed in the reactor during milling. After 30 min at 1000 rpm, incomplete conversion into the desired PCA-OXA cocrystal was observed (see ESI, Figure S6). PXRD analysis of the reaction mixture revealed residual PCA, indicated by its characteristic reflections when compared to the PCA reference. Increasing the milling speed to 1200 and 1500 rpm for 30 min each did not result in complete conversion, and PCA reflections were still observed in the PXRD patterns.^4a^ The incomplete conversion, despite the high milling speed, was attributed to the presence of partially hydrated oxalic acid. Since oxalic acid readily forms the dihydrate as the most stable crystalline form under ambient storage conditions, the effective mass ratio of the reactants was altered, resulting in insufficient stoichiometric amounts of anhydrous oxalic acid required for complete cocrystallization, thereby leaving unreacted PCA. The presence of the dihydrate in the batch used for these experiments was confirmed by PXRD (see ESI, Figure S6). Upon replacing the OXA with a freshly obtained anhydrous batch, full conversion of PCA to the cocrystal was achieved by milling both reagents at 1000 rpm for 30 min, as confirmed by the characteristic PXRD reflections at 15.5, 18.9, 21.7, 27.7, 34.2, and 35.6° 2θ (Figure 4). Owing to the design of the attritor mill, the cocrystal product could be recovered without disassembling complex internal reactor. For product recovery, a coarse sieve was attached to the outlet located at the bottom of the vessel and the reactor was operated under milling conditions. During this process, the powdery product passed through the sieve and was collected in sample bottles, while the grinding media were retained inside the reactor. Milling for 15 min at 1000 rpm resulted in recovery of 96% of the desired cocrystal, highlighting the suitability of the attritor mill for efficient product recovery in mechanochemical processes, even at larger scales.

**Figure 4:**
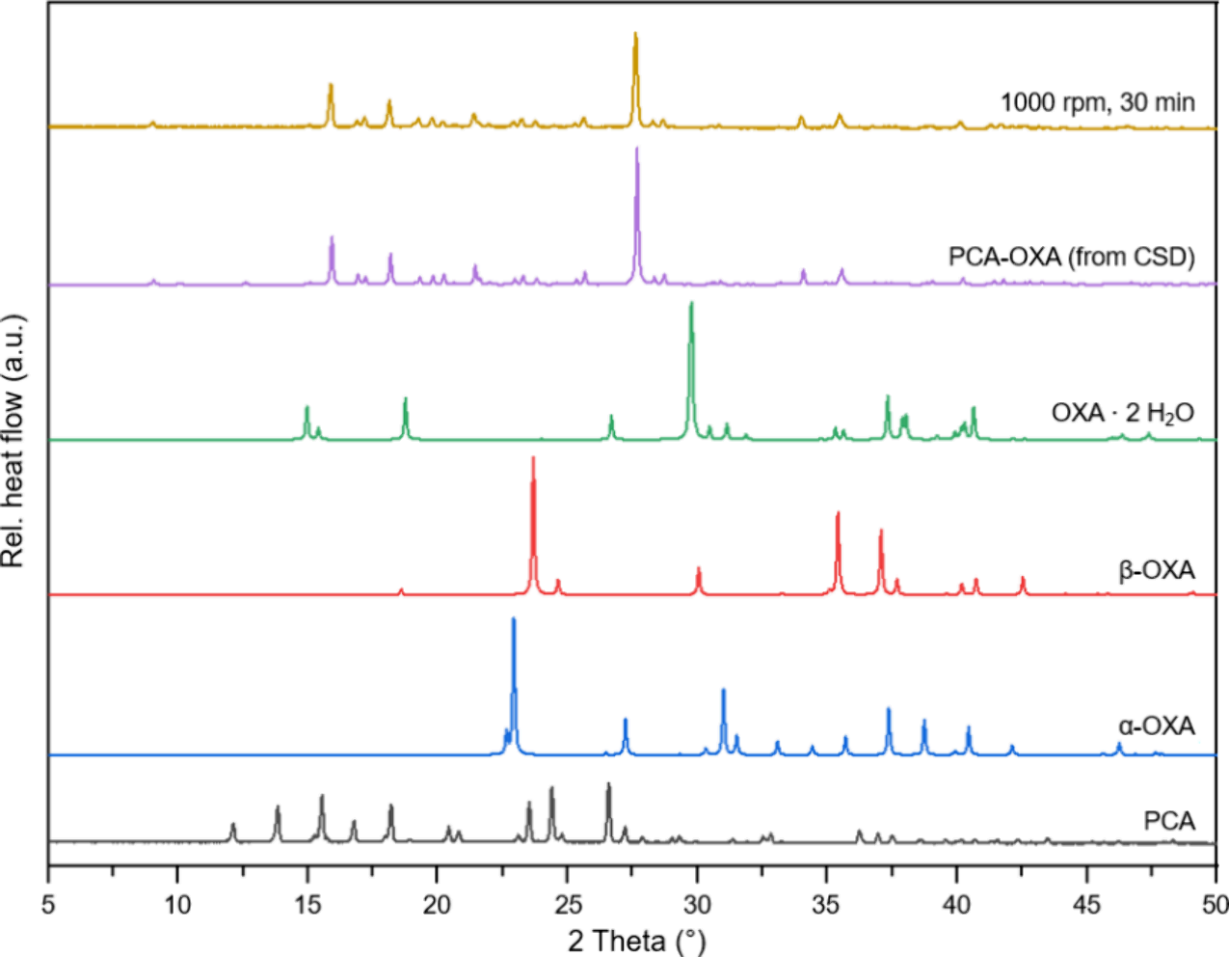
PXRD pattern of PCA (black), calculated α-OXA (blue, CSD 929767), β-OXA (red, CSD 1226343), OXA·2 H_2_O (green, CSD 2042674), and PCA-OXA cocrystal (purple, CSD 720368) as obtained from the CSD. Measured PXRD pattern of PCA-OXA (yellow) after milling PCA and anhydrous OXA at 1000 rpm for 30 min in an attritor mill.

These results demonstrate that an attritor mill can efficiently facilitate cocrystallization at a larger scale than the previously used planetary mills, while also reducing the reaction time from 3–4 h to 30 min due to increased milling speeds. Considering that this technology can be further scaled using industrial mills with larger reactor sizes, attritor milling was identified as a promising approach for achieving pharmaceutical cocrystallization on larger scales. Moreover, these machines can be operated in semicontinuous or continuous modes, making them even more appealing for industrial processes.

### Scale-Up to Kilogram Quantities Using a Drum Mill

While attritor mills are highly promising for pharmaceutical cocrystallization in the multigram to multikilogram scale, further scale-up requires equipment capable of processing substantially larger quantities. Drum mills, widely used in industry and capable of handling throughputs on the order of multitons, provide a suitable platform for such scaling.^17^ To explore the next step under controlled conditions, the cocrystallization of PCA and OXA was investigated in a model drum mill (TM300 from Retsch), bridging the transition from hundred-gram to kilogram-scale processing. This drum mill is a laboratory and pilot-scale device designed for grinding, mixing, and homogenizing of bulk materials. It consists of a rotating cylindrical drum that can be operated in horizontal position. During operation, the rotation of the drum causes the media to lift and fall, producing impact and friction forces that reduce particle size and promote chemical reactions.^14^

An initial experiment was carried out in a 5 L stainless-steel reactor using a batch of 250 g PCA and 150 g OXA. The reaction was initially conducted under neat milling (NM) conditions with 5 kg of 10 mm stainless-steel balls (BPR = 12.5). Samples were collected every 30 min to monitor the reaction progress by PXRD. Under these conditions, the reactants formed a dense, hard layer along the vessel walls, which hindered effective mixing and shielded unreacted material beneath, resulting in incomplete conversion (Figure 5 and 6). The adhered material had to be manually dislodged during sampling using a hammer and flat-headed chisel, adding unnecessary complexity and limiting reproducibility. After 60 min of milling, the material continued to adhere to the vessel walls and complete conversion was not achieved (Figure 5a–c and 6).

**Figure 5:**
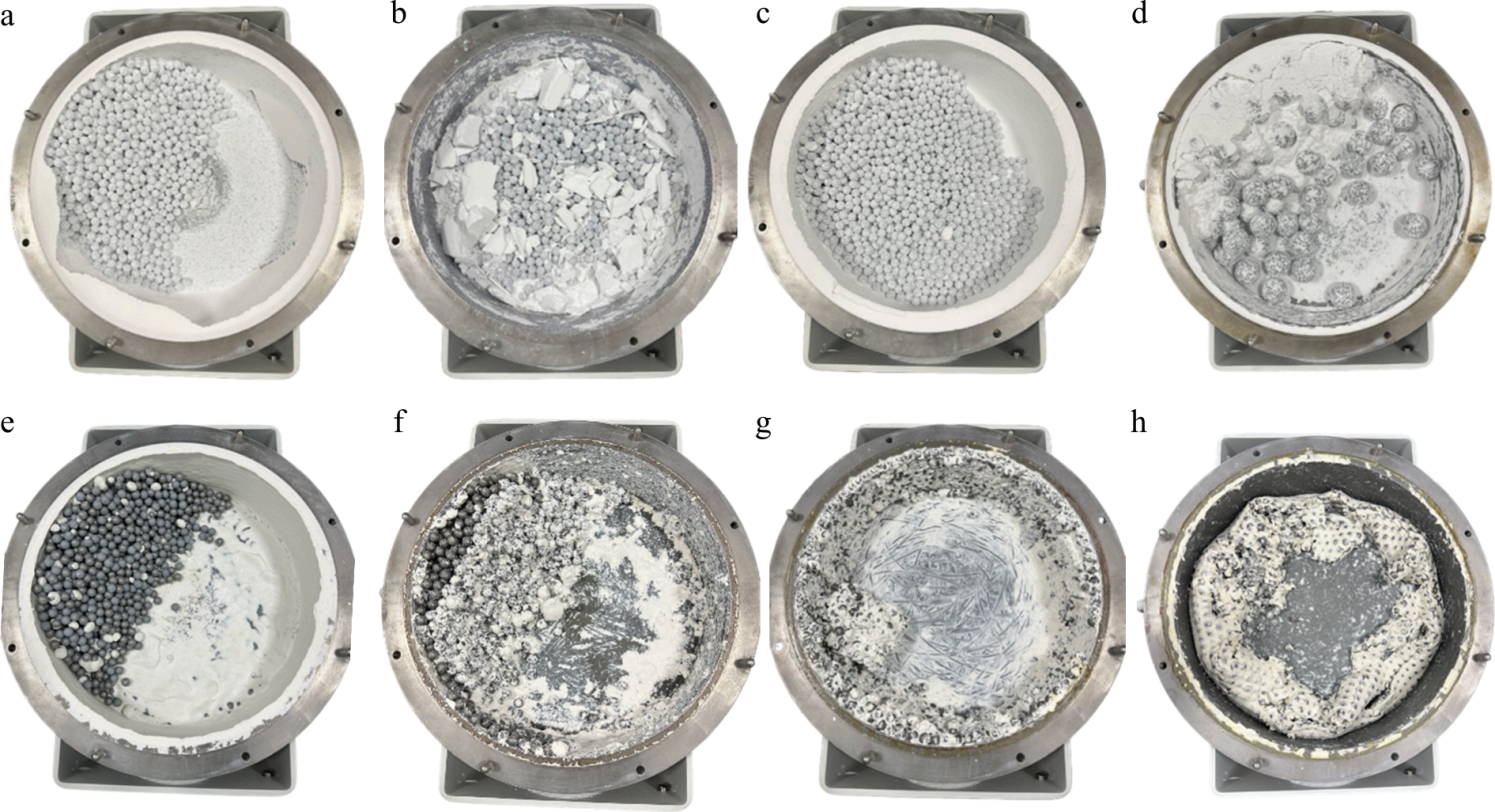
Texture of the reaction mixture for the PCA-OXA synthesis under various conditions: **a)** Caking effect on the walls of the mill after 30 min of dry milling with 5 kg of 10 mm balls. **b)** Layer chiseled down before proceeding to mill further (repeated after every sample collection, this picture is after 30 min of dry milling). **c)** After 60 min of dry milling with 5 kg of 10 mm balls. **d)** Texture of reaction mixture after 30 min of milling with 10 kg of 30 mm balls. **e)** After 30 min of LAG milling with 5 kg of 10 mm balls (η = 0.1 μL/mg). **f)** After 90 min of LAG milling (η = 0.3 μL/mg). Hard layers on the walls seem to have stopped from forming. **g)** After 150 min of LAG milling (η = 0.5 μL/mg). **h)** After 6 h of overnight milling in addition to the overall 4 h of milling. Constituents became pasty and yellowish.

**Figure 6:**
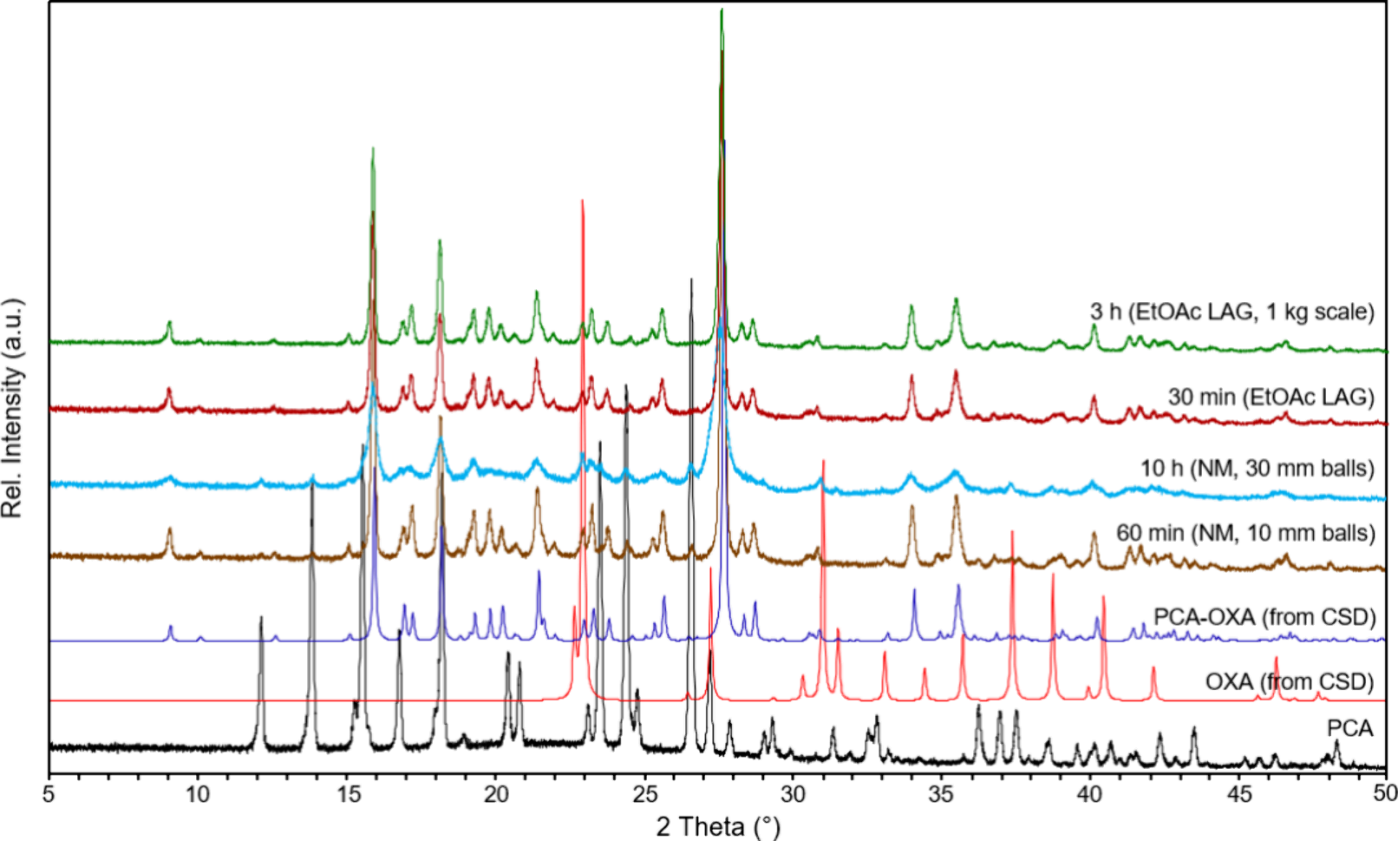
PXRD patterns of PCA-OXA obtained from mechanochemical experiments performed in a drum mill under various conditions. Patterns are shown from bottom to top as follows: simulated PXRD pattern of pure PCA (black) used as a reference; simulated PXRD pattern of OXA (red) and of the PCA-OXA cocrystal (dark blue), both taken from the Cambridge Structural Database (CSD); PXRD pattern of a 250 g scale reaction under neat milling (NM) conditions using 5 kg of 10 mm balls in a 5 L reactor (brown); PXRD pattern of a 250 g scale reaction under NM conditions using 10 kg of 30 mm balls in a 5 L reactor (light blue); PXRD pattern of a 250 g scale reaction under liquid-assisted grinding (LAG) conditions with EtOAc (η = 0.2 mL/g) using 10 kg of 30 mm balls in a 5 L reactor (dark red); PXRD pattern of a 1 kg scale reaction under LAG conditions with EtOAc (η = 0.2 mL/g) using 15 kg of 30 mm balls in a 14 L reactor (green). Representative reflections are observed at 2θ = 9.0°, 10.0°, and 27.8° for PCA-OXA, at 12.1° and 13.9° for PCA, and at 31.0° for OXA.

To improve the texture and mobility of the reaction mixture, a second batch was milled under neat conditions using larger milling balls (30 mm diameter) and a total ball mass of 10 kg, increasing the BPR to 25. This modification successfully prevented material adhesion, and a freely flowing powder was observed after 30 min of milling (Figure 5d). However, conversion remained incomplete even after extended milling for 10 h, as PXRD analysis revealed a fraction of unreacted starting materials (Figure 6 and Figure S7).

Given the incomplete conversion observed in previous drum mill experiments (Figures 5 a–d and 6), the use of liquid-assisted grinding (LAG) was explored.^10^ In the first experiment, with 5 kg of 10 mm stainless-steel milling balls, EtOH was introduced after 90 min of dry milling at a solvent-to-solid ratio of η = 0.1 mL/g. However, this small amount of EtOH did not prevent the formation of the hard surface crust previously observed on the vessel walls (Figure 5e). Increasing the EtOH content to η = 0.3 mL/g improved the texture of the reaction mixture, preventing the formation of adherent layers on the vessel walls (Figure 5f). Notably, after 6 h of continued milling under these conditions, the reaction mixture transformed into a pasty, yellowish mass (Figure 5h). Nuclear magnetic resonance (NMR) analysis of the reaction mixture revealed that oxalic acid had undergone esterification with the LAG solvent EtOH, forming diethyl oxalate (see ESI, Figure S8).

Interestingly, this side reaction was not observed during the preliminary small-scale experiments, nor has it been reported in earlier studies on PCA-OXA cocrystal formation using alcoholic LAG solvents.^4a^ While we cannot fully exclude the possibility that trace amounts of diethyl oxalate were formed at smaller scales but remained below the detection limit, these quantities were insufficient for analytical characterization. This observation highlights an important aspect of mechanochemical scale-up. Processes that appear robust on the milligram or gram scale can exhibit new behaviors when transferred to larger scales. Increased material volumes and higher cumulative energy input can promote side reactions that remain undetectable at smaller scales, where quantities of side products are often insufficient for detailed analytical characterization. Furthermore, macroscopic texture changes are more pronounced and easily noticeable in larger batches, providing additional insights into process dynamics that are not always apparent in small-scale trials.

Further experiments were performed using LAG, focusing on solvents that would not chemically react with the reactants and that are suitable for scale-up. In this context, EtOAc was selected for subsequent milling experiments. Repeating a batch with 10 kg of 30 mm milling balls inside a 5 L vessel with 250 g of PCA and 150 g of OXA and the addition of EtOAc (η = 0.2 mL/g) enabled complete cocrystallization within 30 min of milling. The solvent was readily removed by leaving the milling vessel open overnight in a fume hood, yielding pure PCA-OXA cocrystals as confirmed by PXRD (Figure 6).

For further scale-up, the reactor volume was increased by employing a 14 L stainless steel reactor. This reactor was charged with 1.0 kg of PCA, 0.6 kg of OXA, as well as 15 kg of 30 mm stainless steel balls (BPR = 9.4). EtOAc (η = 0.2 mL/g) was used as the LAG solvent. Complete conversion was achieved within 3 h as determined by PXRD (Figure 6). Compared to the smaller-scale experiment, which reached completion in only 30 min, the extended milling time required for the larger batch can be attributed to several factors. First, the increased reactor volume leads to a lower frequency of effective ball and powder collisions per unit mass of material. This effect is further reflected in the lower BPR at larger scale (9.4 at 1 kg scale vs 25 at 250 g scale), resulting in reduced energy input per mass of reactant. Moreover, the higher sample mass reduces the relative energy imparted to individual particles and the larger fill level of milling balls and powder alters the dynamics of the grinding media, resulting in less efficient mixing and a longer diffusion path for LAG solvent to uniformly wet all particles. Upon completion of the reaction, the milling media were separated using a coarse sieve, yielding 1.51 kg (95%) of the final PCA-OXA cocrystal product.

### Comparison of Mechanochemical Technologies for Scale-Up

Each technology investigated for the synthesis and scalability of PCA-OXA cocrystals offers distinct advantages and limitations with respect to reaction time, achievable scale, operational simplicity, and product recovery (Table 1). Planetary ball mills served as the primary screening and optimization platforms for the PCA-OXA cocrystallization. Owing to their high energy input, these mills enabled rapid cocrystal formation at the laboratory scale, with complete conversion typically achieved within a few hours, depending on jar volume, ball-to-powder ratio, and milling speed. The well-defined and reproducible milling conditions of planetary mills make them particularly suitable for mechanistic studies and parameter optimization. However, the practical scalability of planetary ball mills is limited. Even when larger vessels are employed, the total processable mass remains restricted and the complex design and movement pattern of the machine makes industrial applications difficult. Consequently, while planetary ball mills are widely available and commonly used in academic laboratories, their applicability for preparative or industrial-scale production is limited.

**Table 1:** Summary of Optimized Scale-Up Conditions for PCA-OXA Cocrystal Synthesis across Different Milling Technologies

parameter/mill	planetary ball mill	attritor mill	drum mill
scale (PCA starting material)	25 g	100 g	1.0 kg
reactor volume	500 mL	1 L	14 L
milling balls	12 × 18 mm (m = 380 g)	5 mm (m = 2 kg)	30 mm (m = 15 kg)
milling speed	250 rpm	1000 rpm	60 rpm
BPR	9.5	12.5	9.4
reaction time	4 h	45 min^a^	3 h
bulk temperature^b^	40–50 °C	18 °C (cooled)	<30 °C
yield	36.9 g (93%)	146.9 g (96%)	1.51 kg (95%)
energy input (per h)	1.73 kWh	1.1 kWh	0.75 kWh
specific energy input (SEI)	187.6 kWh/kg	5.62 kWh/kg	1.49 kWh/kg

a 30 min reaction time and 15 min out outgrinding.

b Temperature measured immediately after the reaction using an IR thermometer.

The attritor mill represents an intermediate step between laboratory-scale planetary mills and larger-scale continuous or semicontinuous milling technologies. In the present study, the Simoloyer enabled efficient cocrystal formation at significantly higher throughput, while maintaining comparatively short reaction times. Once suitable milling parameters are established, complete cocrystallization could be achieved within tens of minutes, demonstrating the high efficiency of energy transfer in this system. A key advantage of the attritor mill is its ability to process larger powder masses with 900 L reactors available in industrial settings. In addition, despite the mechanically complex internal reactor design, product recovery can be achieved without disassembling the reactor, using an integrated outlet and sieve system. This feature considerably simplifies workup, minimizes material losses, and enables the development of sequential batch processing. Attritor mills are well established in industrial settings, particularly in metallurgy and materials processing, which underscores their relevance for further scale-up and potential industrial implementation of mechanochemical cocrystal synthesis.

The drum mill offers a fundamentally different milling regime, characterized by lower energy input and longer reaction times. In contrast to planetary and attritor mills, cocrystal formation in the drum mill required substantially extended milling durations or the use of LAG techniques, reflecting the gentler mechanical treatment. Nevertheless, the drum mill enables processing of comparatively large material quantities and provides excellent powder mixing over extended time scales. From an operational perspective, the drum mill is simple in design, easy to operate, and well suited for long-term milling experiments. Product recovery is straightforward, as the reactor can be emptied easily without complex disassembly. While drum mills are less commonly used for rapid mechanochemical transformations, they are attractive for scale-up scenarios where mild milling conditions, robustness, and simplicity are prioritized over reaction speed. Drum mills are also more closely aligned with equipment already used in certain industrial sectors, which may facilitate technology transfer.

Abrasion of milling media and reactor materials is an important consideration in mechanochemistry, particularly at larger scales and longer milling times. While a detailed investigation of trace metal contamination is beyond the scope of this study, the expected relative abrasion of different milling devices can be discussed qualitatively. In horizontal high-energy attritor mills, abrasion is typically the highest due to the combination of high BPR and very high rotational speeds (up to 1800 rpm), leading to increased contact and friction. Planetary ball mills generally exhibit moderate abrasion, as friction forces are elevated but can be typically controlled by adjusting the BPR and milling conditions. Drum mills generally show lower abrasion, even at higher BPR, due to their relatively low rotational speeds, which typically range from 20 to 60 rpm. These differences may influence potential trace metal contamination levels in the products and should be considered when designing scale-up protocols.

Another parameter to consider is the energy consumption of the different milling devices. For the specific models used in this study, the planetary ball mill consumes 1.73 kWh for a 1 h reaction at 250 rpm, corresponding to a specific energy input of 187.6 kWh per kilogram of product. The attritor mill requires 1.1 kWh at 1000 rpm for the same reaction duration, resulting in a lower specific energy input of 5.62 kWh/kg. The drum mill is the most energy-efficient device for this cocrystallization, consuming only 0.75 kWh at 60 rpm for a 1 h reaction, which corresponds to 1.49 kWh per kilogram of product.

While the present study focuses on the PCA-OXA system as a model case, the insights gained are expected to extend beyond this specific example. In particular, the identification of scale-dependent factors such as energy input, BPR, and rheological behavior provides a general framework for the scale-up of mechanochemical processes. Although the absolute values of these parameters will vary depending on the physicochemical properties of the system under investigation, the qualitative trends observed here (such as changes in energy transfer efficiency, heat generation, and the potential emergence of side reactions under liquid-assisted grinding conditions) are likely to be broadly applicable. Consequently, this work contributes to a more general understanding of mechanochemical scale-up and may serve as a guideline for the development of scalable processes for other cocrystal systems and solid-state transformations.

## Conclusion

The scale-up of PCA-OXA cocrystals was systematically investigated across multiple scales and milling technologies. Planetary ball mills with jar volumes ranging from 12 to 500 mL were suitable for the preparation of multigram quantities of PCA-OXA. Transitioning from initial batches of 0.5–2.5 g in a FRITSCH Pulverisette 7 planetary ball mill to 25 g in a FRITSCH Pulverisette 5 mill proceeded without major issues, likely due to the similarity of the equipment.

For hundred-gram quantities, an attritor mill was employed, achieving full conversion of PCA into PCA-OXA within only 30 min at higher speeds of 1000 rpm and a product recovery of 96% without the need for disassembling the reactor. Challenges emerged when scaling up to kilogram quantities using a drum mill. While neat milling was effective in planetary and attritor mills, liquid-assisted grinding was required in the drum mill to achieve complete conversion and prevent formation of a hard crust on the reactor walls. Careful selection of the LAG solvent was essential, as EtOH reacted with oxalic acid to form diethyl oxalate, a side reaction not observed at smaller scales. Ultimately, the addition of small amounts of EtOAc as LAG enabled the recovery of 1.51 kg (95%) of PCA-OXA from 1 kg of PCA and 0.6 kg of OXA after 3 h of milling. These results provide a better understanding of the opportunities and limitations associated with mechanochemical devices for the gradual scale-up of pharmaceutical cocrystals, highlighting both scale-dependent challenges and strategies to maintain product quality across orders of magnitude in batch size.

## Supplementary Material



## Notes

Detailed experimental procedures, cleaning protocols, and analytical data (PDF)

## ABBREVIATIONS

API: active pharmaceutical ingredient
PCA: paracetamol
OXA: oxalic acid
CIT: citric acid
THP: theophylline
PHE: phenazine
TMG: trimethylglycine
PCA-OXA: paracetamol-oxalic acid cocrystal
BPR: ball-to-powder ratio
WHO: World Health Organization
NM: neat milling
LAG: liquid-assisted grinding
DSC: differential scanning calorimetry
PXRD: powder X-ray diffraction
NMR: nuclear magnetic resonance
P7: FRITSCH Pulverisette 7 planetary ball mill classic line
P5: FRITSCH Pulverisette 5 planetary ball mill classic line.
