# Supplementary material for: Systematic Scale-Up of Mechanochemical Paracetamol–Oxalic Acid Cocrystal Synthesis across Multiple Milling Technologies
Source: Org Process Res Dev. 2026 May 13;30(6):1615–24. doi: 10.1021/acs.oprd.6c00052 (PMC13289493; doi:10.1021/acs.oprd.6c00052)
Supplement: Supplementary file 1 [file op6c00052_si_001.pdf]

Supporting Information

for

Systematic Scale-Up of Mechanochemical Paracetamol−Oxalic Acid Co-Crystal Synthesis Across Multiple Milling Technologies

Jan-Hendrik Schöbel, Dhyanesh Gopinath, and Michael Felderhoff*

Department of Heterogeneous Catalysis, Max-Planck-Institut für Kohlenforschung, Kaiser-Wilhelm-Platz 1, D-45470 Mülheim an der Ruhr

*E-Mail: felderhoff@kofo.mpg.de

Table of Content

Section **Page**

1. General Information S2
2. Experimental Procedures S3
3. Equipment Cleaning S5
4. Analytical Data S6
5. References S12

1. General Information

All chemicals were obtained from commercial suppliers and used without further purification. Solvents for liquid-assisted grinding (LAG) and cleaning purposes were of technical grade.

Mechanochemical experiments were performed using the following instruments: Fritsch Pulverisette 7 classic line (planetary ball mill), Fritsch Pulverisette 5 classic line (planetary ball mill), Zoz Simoloyer CM01 (attritor mill), and Retsch TM 300 (drum mill). Milling jars and balls were made of stainless steel (ρ = 7.9 g/cm^3). Samples were dried overnight in a fume hood and ground into fine powders using an agate mortar and pestle prior to analysis.

Nuclear magnetic resonance (NMR) spectra were recorded on a Bruker-300 Ultrashield spectrometer using deuterated chloroform ($CDCl_3$) as solvent. Chemical shifts (δ) are reported in parts per million (ppm) and are referenced to the residual non-deuterated solvent signal. Raw data were processed with MestReNova software.

Differential scanning calorimetry (DSC) analyses were performed using a METTLER TOLEDO DSC820 instrument with a heating rate of 10 °C/min and a scanning range of either 80 °C to 300 °C or 50 °C to 250 °C, both under an air purge flow of 50 mL/min. Samples (6–10 mg) were placed in standard 40 µL aluminium crucibles and sealed with aluminium lids (with or without pinhole) using a METTLER TOLEDO stamp.. Data were processed using STARe software from METTLER TOLEDO and Origin 2019b from OriginLab Corporation.

Powder X-ray diffraction (PXRD) measurements were performed using a STOE Powder Diffraction System that contained a sealed long-fine-focus Cu-tube operating at 40 kV and 40 mA, a short collimator, a curved Ge (111) monochromator to yield pure Cu Kα1 (λ = 1.54056 Å) radiation. Detection was performed using a linear position-sensitive detector (PSD) filled with a CH_4/Ar (90:10) mixture. Each sample was loaded into a single 0.7 mm borosilicate glass capillary and measured between the range of 2° and 50° (2θ) at a scanning rate of 40 s per step. Data processing and editing were performed using STOE Win XPOW V3.05 software. PXRD was used as the primary method to assess phase formation and conversion during mechanochemical processing. While this technique is well suited to identify crystalline phases, it is inherently limited in its ability to detect low levels of organic impurities or amorphous content. Consequently, trace organic impurities cannot fully be excluded. Similarly, amorphous fractions, which do not exhibit long-range order, are not readily detected by PXRD. Although the presence of sharp and well-defined diffraction peaks indicates a predominantly crystalline material, minor amorphous contributions cannot be ruled out.

Approximate bulk temperatures were determined using a Fluke 64 MAX infrared (IR) thermometer. Measurements were taken at various positions within the milling jar (bottom, inner walls, lid, and milling balls) and the highest recorded temperature was reported.

2. Experimental Procedures

For the initial co-former screening reactions, PCA (500 mg, 3.31 mmol) was placed in a 12 mL stainless steel jar together with 25 stainless steel milling balls (d = 5 mm). Afterwards, the corresponding mass of the co-former was added (OXA: 298 mg; CIT: 318 mg; PHE: 1.19 g; THP: 298 mg; TMG: 388 mg). Where indicated, EtOH was added as LAG solvent (η = 0.2 μL/mg). The reactions were conducted at room temperature at a frequency of 600 rpm in a Fritsch P7 planetary ball mill for 1 h.

For PCA-OXA screening and upscaling reactions, different mills and reactor sizes were employed:

P7 planetary mill (12 mL vessel): 500 mg of PCA and 298 mg of OXA were added along with 50 milling balls (5 mm diameter). Milling was carried out at 650 rpm for 4 h. The product was manually recovered from the milling jar using a spatula.

P7 planetary mill (45 mL vessel): 500 mg of PCA and 298 mg of OXA were added along with 50 milling balls (5 mm diameter). Milling was performed at 650 rpm for 4 h. The same conditions were applied for the screening of 2.5 g of PCA and 1.5 g of OXA. The product was manually recovered from the milling jar using a spatula.

P5 planetary mill (500 mL vessel): 5.0 g of PCA and 3.0 g of OXA were added together with 12 milling balls (18 mm diameter). Milling was performed at 250 rpm for 2 h, with samples collected every 30 min. For the screening of 25 g of PCA and 15 g of OXA, the reagents were milled at 250 rpm for 4 h using 12 x 18 mm balls. An additional experiment was carried out using 24 milling balls (10 mm diameter) while all other parameters were kept constant. The product was manually recovered from the milling jar using a spatula.

Attritor mill (1 L vessel): 100 g of PCA and 59.6 g of OCA were added to a 1 L stainless steel reactor containing 2 kg of stainless steel balls (d = 5 mm). The mixture was milled at 1000 rpm for 0.5 h in an attritor mill (model: Simoloyer CM01 from Zoz GmbH) equipped with a cooling jacket, maintaining a reactor temperature of 18 °C. The material was recovered by milling it

out through the reactor’s bottom outlet, with a coarse sieve retaining the milling balls while the powder was collected. The outgrinding was performed for 15 min at 1000 rpm.

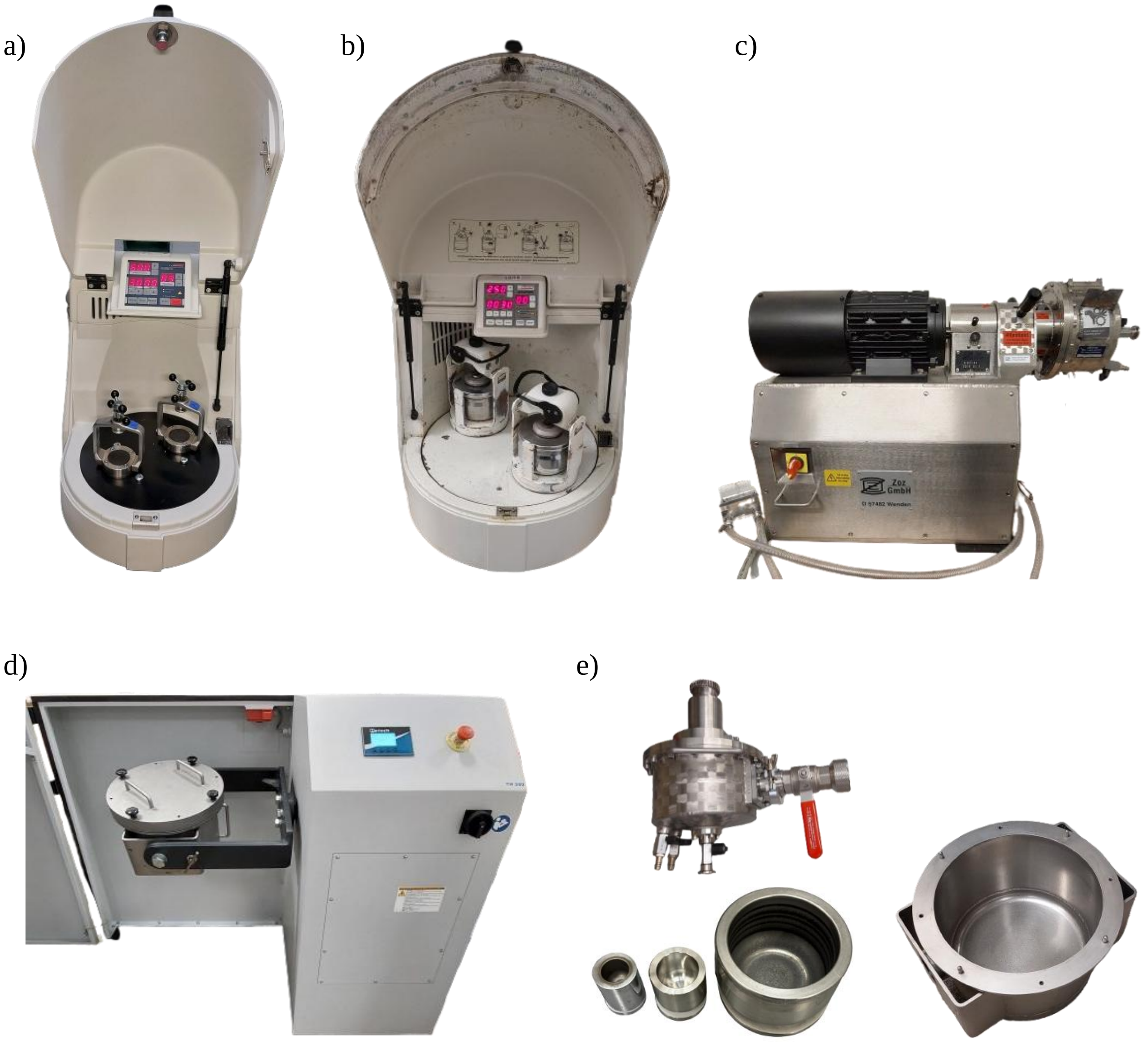

Figure S1. Mills and reactors used in this study: a) Fritsch Planetary Ball Mill PULVERISETTE 7 (P7) classic line with two working stations; b) Fritsch Planetary Ball Mill PULVERISETTE 5 (P5) classic line with two working stations and attached 500 mL stainless steel jars; c) Zoz Simoloyer CM01 with attached 1 L stainless steel reactor; d) Retsch TM300 drum mill with attached closed 14 L reactor; e) various milling jars and reactors for the mills mentioned above (12 mL, 45 mL, 500 mL for planetary mills; 1 L reactor with adapter for cooling and product recovery for the Simoloyer; 14 L stainless steel reactor for the drum mill).

TM300 drum mill (5 L vessel): 250 g of PCA and 149 g of OXA were added along with 10 kg of milling balls (30 mm diameter). Milling was performed at 60 rpm for 10 h. EtOAc (80 mL; η = 0.2 μL/mg) was added after 10 h, and milling was continued for an additional 30 min. The grinding media were removed by filtration through a coarse sieve.

TM300 drum mill (14 L vessel): 1.0 kg of PCA and 600 g of OXA were added along with 15 kg of milling balls (30 mm diameter). Milling was carried out at 60 rpm for 3 h using EtOAc (80 mL; η = 0.2 μL/mg) as LAG solvent. The grinding media were removed by filtration through a coarse sieve.

3. Equipment cleaning

Planetary ball mills: After recovering the product manually and separating the grinding material, the milling balls and jars were cleaned with water and acetone. Subsequently, the jars were milled with sand at 600 rpm until all residual material and surface impurities were removed.

Attritor mill: After discharging the product through the outlet at the bottom of the machine by outgrinding at 1000 rpm for 15 min, while retaining the milling media with a coarse sieve, the milling balls and jars were cleaned with water and acetone.

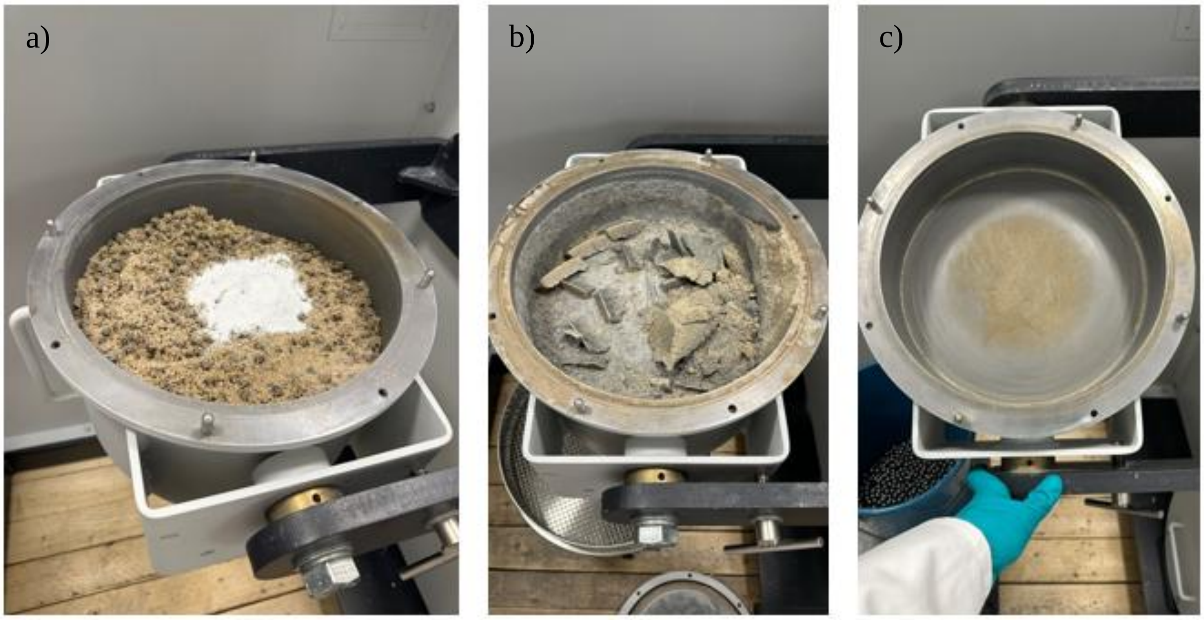

Figure S2. Cleaning procedure of the drum mill after performing the PCA-OXA co-crystal synthesis: a) 14 L reactor loaded with sand (quartz silicon dioxide, 50-70 mesh particles size) and sodium carbonate; b) reactor after milling at 60 rpm for 1 h; c) reactor after removal of the cleaning materials, showing the restored inner surface.

Drum mill: After each experiment, the milling balls were first collected and then washed with water followed by acetone. The reactor, containing residual product, was then charged with the cleaned milling balls, sodium carbonate to neutralize any remaining oxalic acid and to remove iron oxides or hydroxides that may have formed, as well as sand to mechanically detach

adhering material. Subsequently, the reactor was milled with sand at 60 rpm until all residual material and surface impurities were removed. Finally, both the reactor and milling media were rinsed with water and acetone (Figure S2).

4. Analytical Data

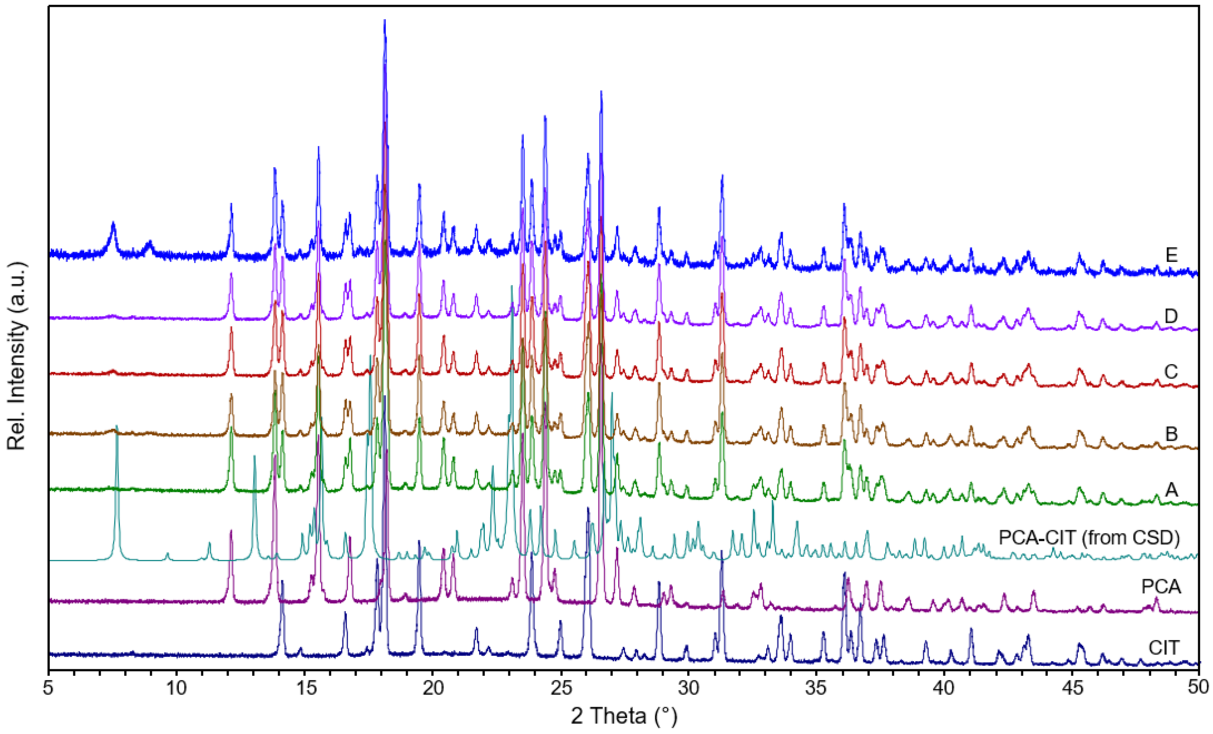

Figure S3. PXRD patterns of PCA-CIT synthesized in a planetary ball mill under various conditions. Patterns are shown from bottom to top as follows: pure CIT (dark blue) and PCA (purple) measured as a reference. PCA-CIT (cyan) taken from the Cambridge Structural Database (CSD) and used as a reference (CSD 803736). Conditions (1:1 molar ratio of PCA-CIT), A (green): 45 mL jar, 15 x 5 mm balls, 600 rpm, 2 h, 500 mg PCA. B (brown): 12 mL jar, 2 x 10 mm balls, 600 rpm, 1 h, 200 mg PCA. C (red): 12 mL jar, 2 x 10 mm balls, 600 rpm, 2 h, 200 mg PCA. D (light purple): 45 mL jar, 5 x 10 mm balls, 600 rpm, 2 h, 500 mg PCA. E (blue): 12 mL jar, 2 x 10 mm balls, 800 rpm, 6 h, 200 mg PCA.

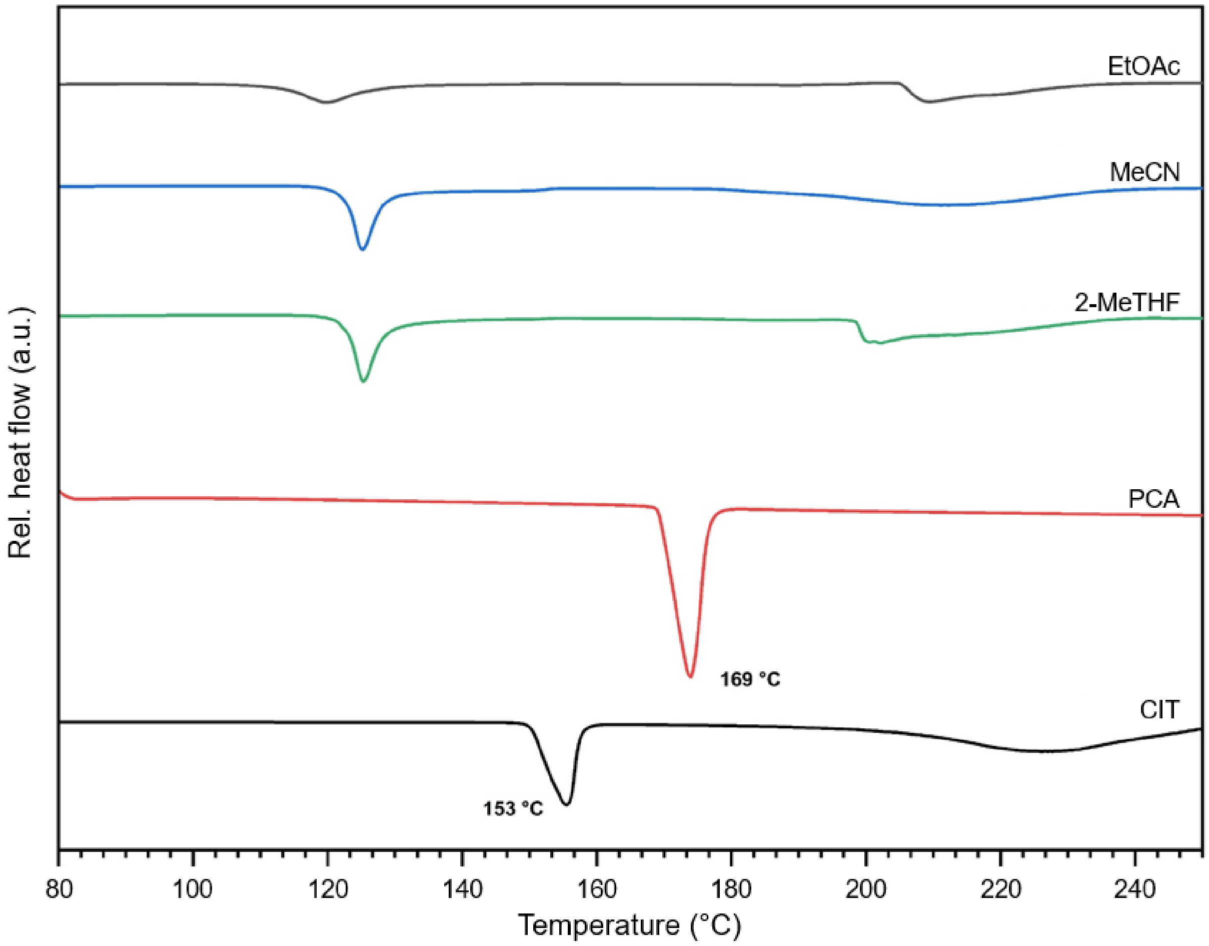

Figure S4. DSC thermograms of PCA-CIT synthesized in a planetary ball mill under liquid-assisted grinding (LAG). Thermograms are shown from bottom to top as follows: pure CIT (black) and PCA (red) measured as a reference. Reaction performed with 2-MeTHF (green), MeCN (blue), or EtOAc (black) as LAG solvents. General conditions: P7, 1:1 molar ratio of PCA:CIT, PCA (200 mg), LAG solvent (η = 0.2 µL/mg), 12 mL jar, 2 x 10 mm balls, 600 rpm, 2 h.

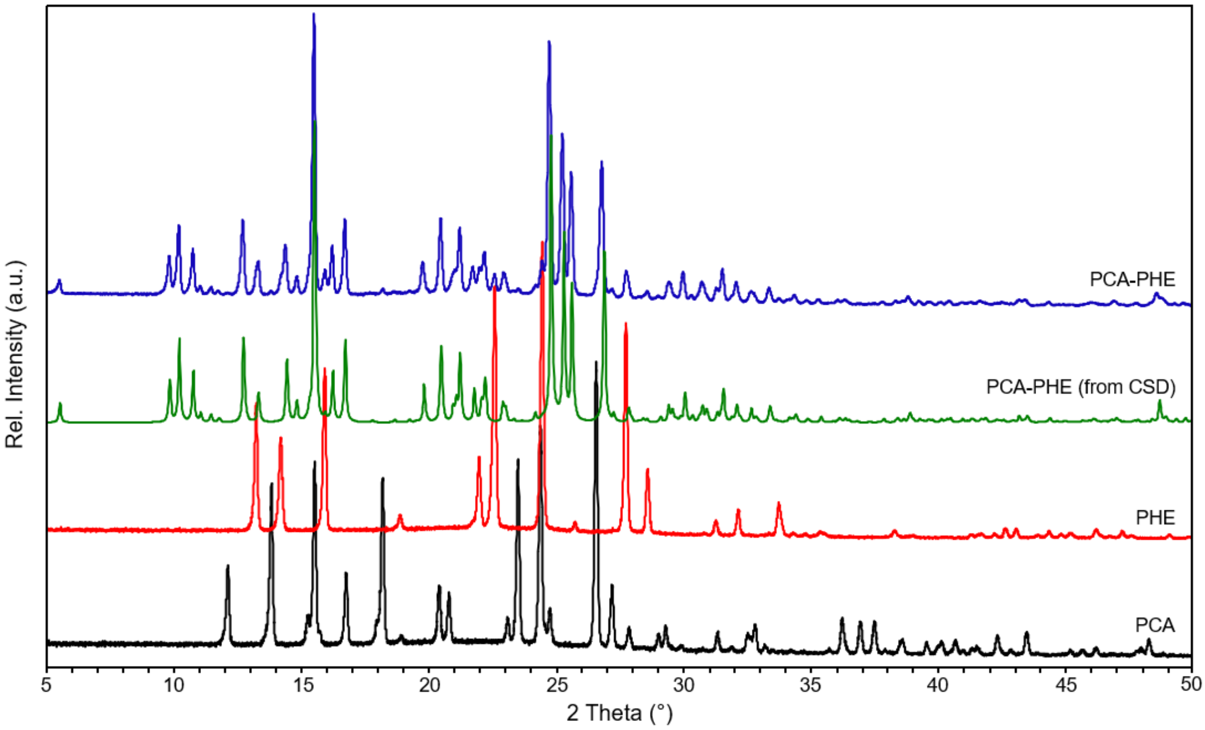

Figure S5. PXRD patterns of PCA-PHE synthesized in P7. Patterns are shown from bottom to top as follows: pure PCA (black) and PHE (red) measured as a reference. PCA-PHE (green) taken from CSD and used as a reference (CSD 720365). PCA-PHE (blue), synthesized with the following conditions: PCA (500 mg), 1:1 molar ratio of PCA:CIT, 45 mL jar, 15 x 5 mm balls, 600 rpm, 2 h.

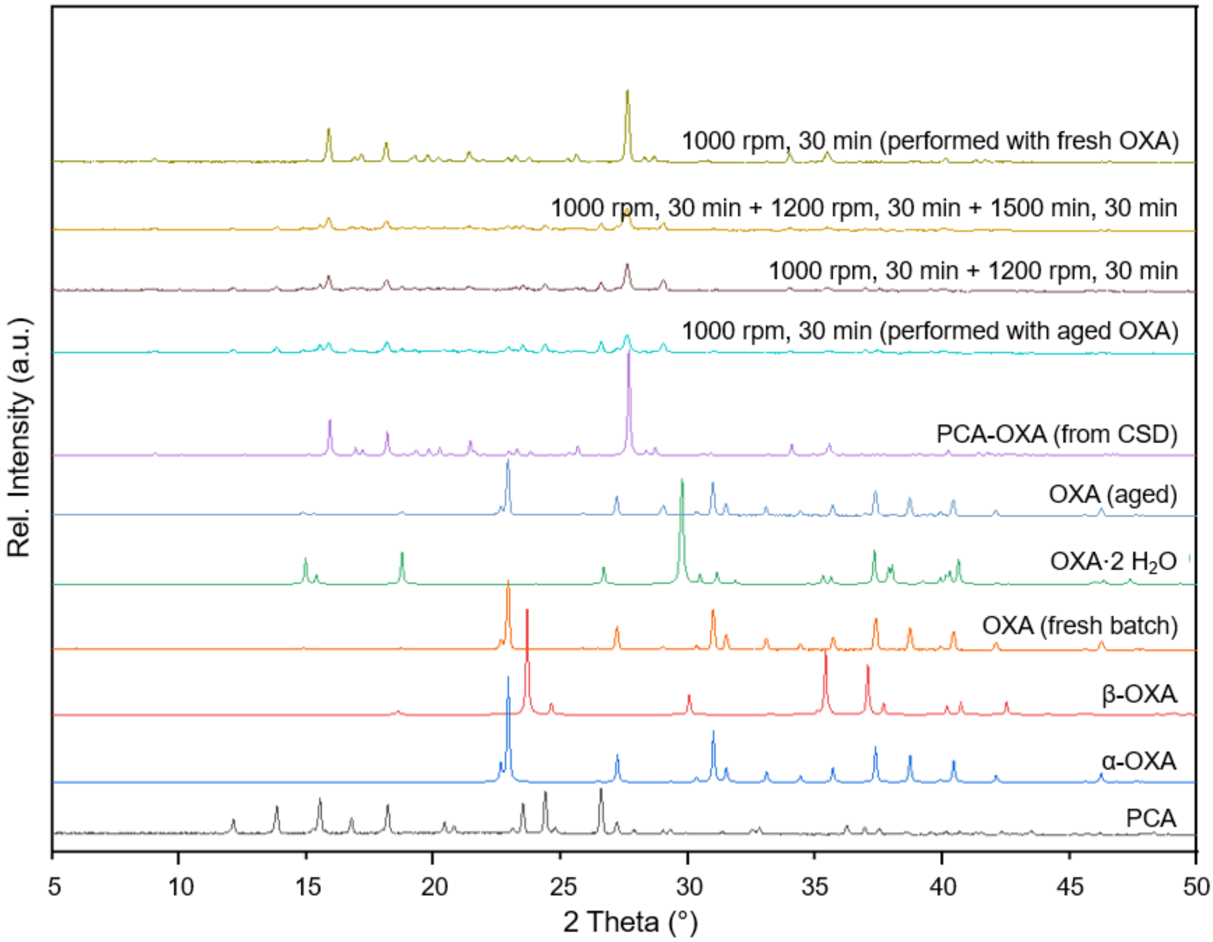

Figure S6. PXRD patterns of PCA-OXA co-crystals obtained after milling under various conditions in an attritor mill. Reference PXRD patterns of α-OXA (blue, CSD 929767), β-OXA (red, CSD 1226343), OXA·2H_2O (green, CSD 2042674), and the PCA-OXA co-crystal (purple, CSD 720368) were obtained from the CDS. Measured PXRD patterns of PCA (black), aged OXA (light blue) stored under ambient conditions for several weeks, and fresh anhydrous OXA (orange) are also shown. Patterns in cyan, brown, and yellow correspond to experiments performed using aged OXA, while the gold pattern corresponds to fresh OXA.

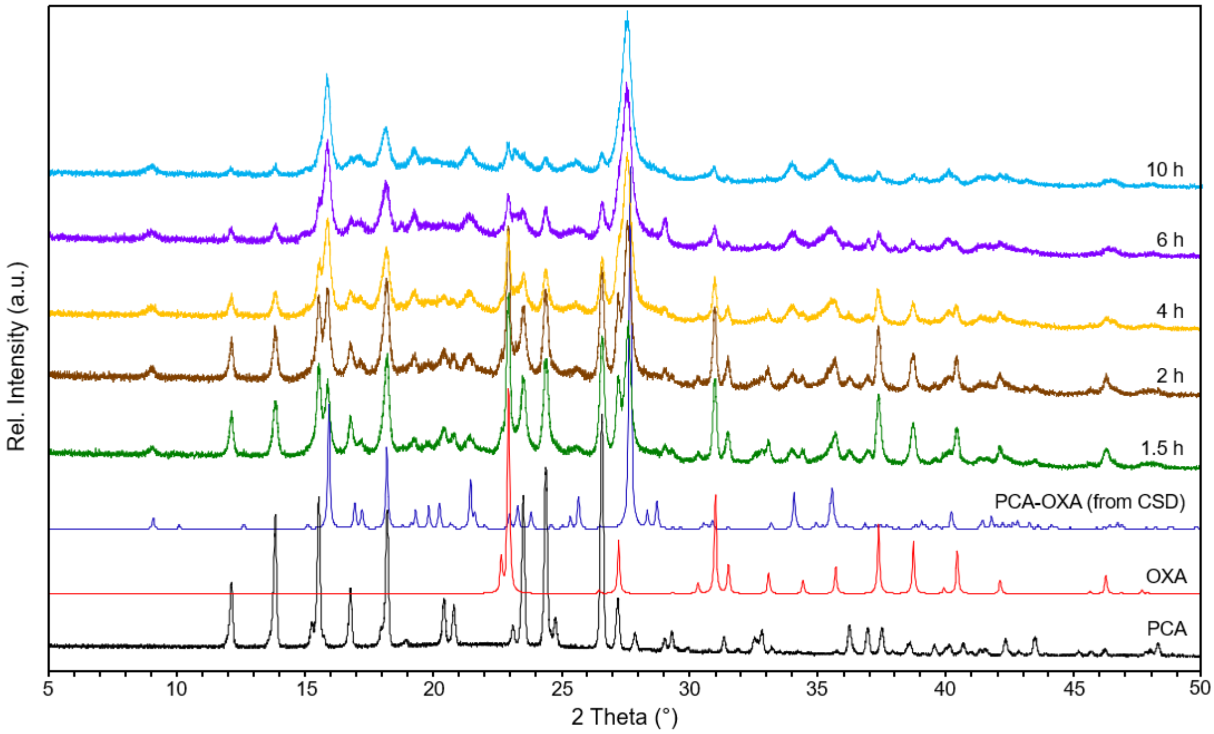

Figure S7. PXRD patterns of PCA-OXA, synthesized in a drum mill with the following conditions: PCA (250 g), 1:1 molar ratio of PCA:OXA, 5 L reactor, 10 kg x 10 mm balls, 60 rpm. Patterns are shown from bottom to top as follows: pure PCA (black) measured as a reference. OXA (red) and PCA-OXA (dark blue, CSD 720368) as obtained from the CSD. PXRD patterns after different time intervals: 1.5 h (green), 2 h (brown), 4 h (yellow), 6 h (purple), 10 h (light blue). Conversion increases with time, but characteristic PCA reflections still remain detectable after 10 h.

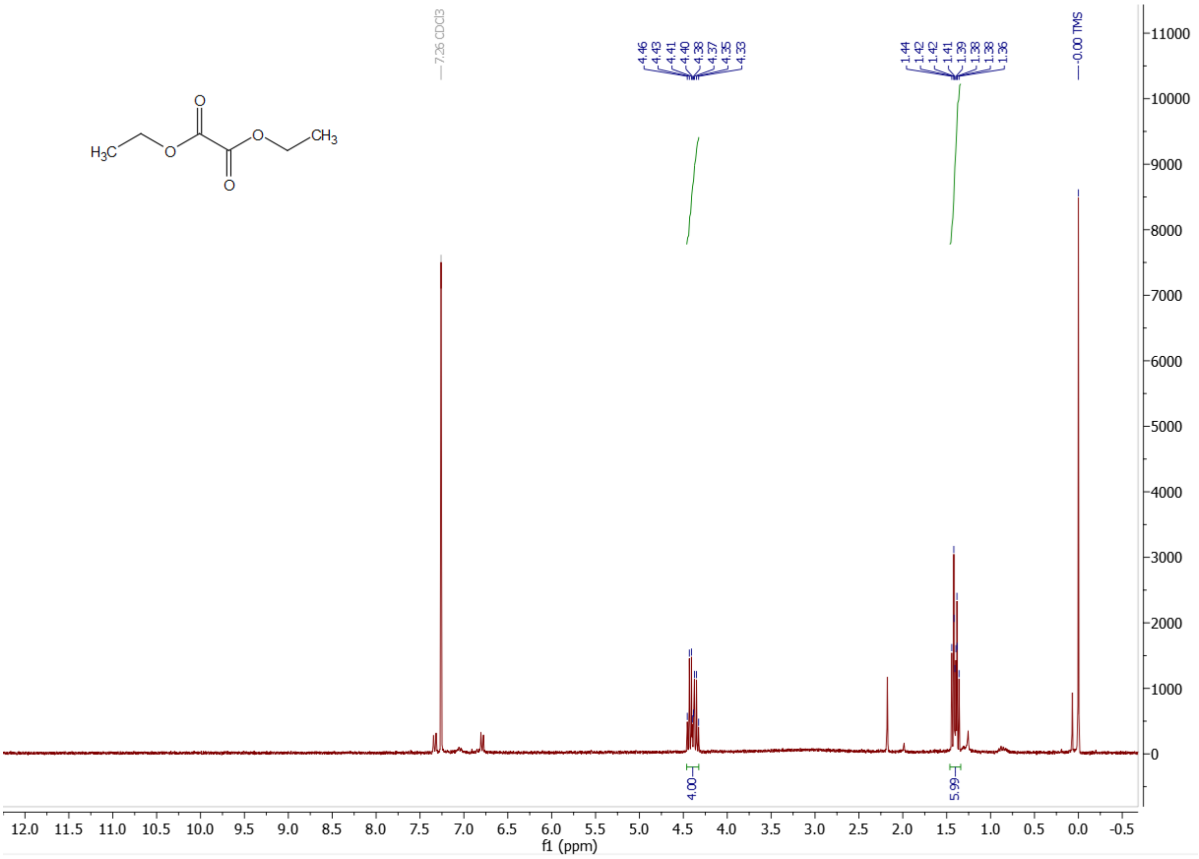

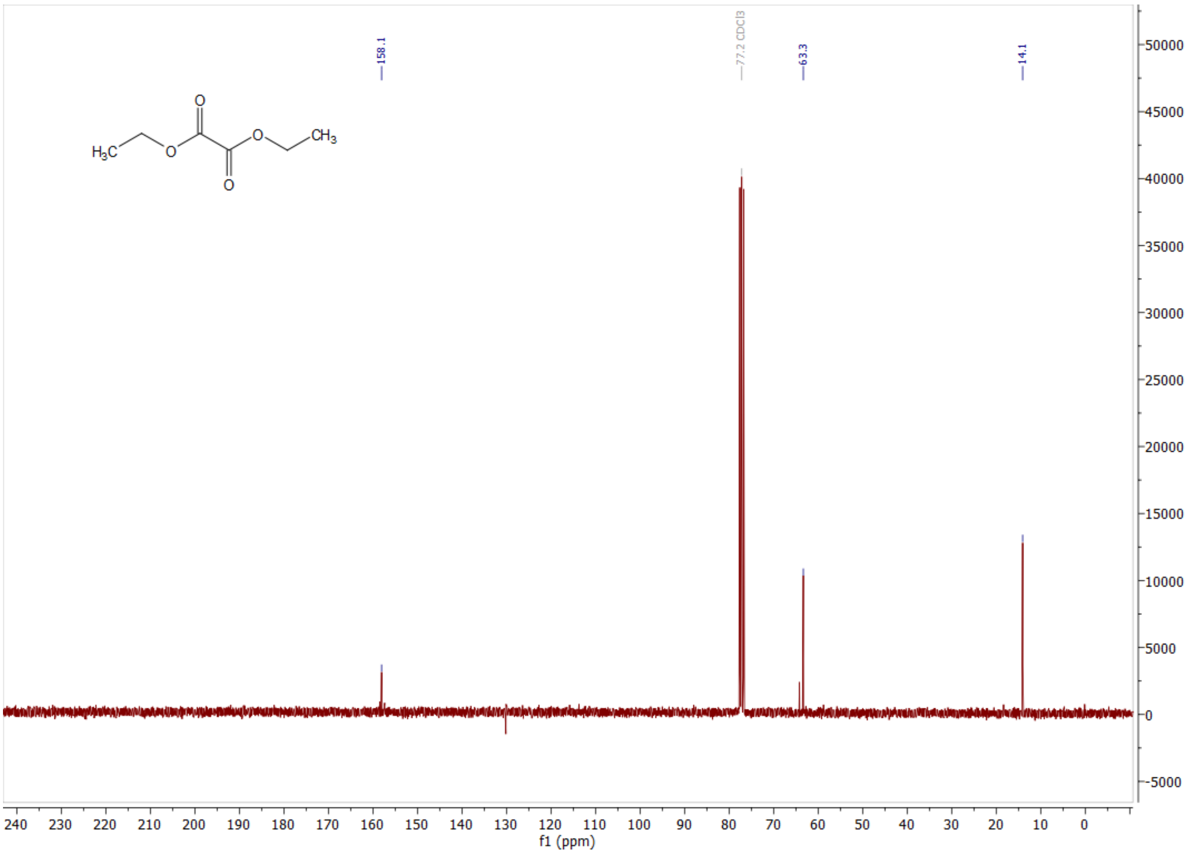

Figure S8. ^{1}H and ^{13}C NMR spectra of diethyl oxalate isolated as a side product from the PCA-OXA synthesis in the drum mill using EtOH as LAG. PCA peaks are only visible in trace amounts due to its low solubility in $CDCl_3$. The suspension was filtered through a PTFE syringe filter prior to NMR analysis. ^{1}H NMR (300 MHz, $CDCl_3$): δ = 4.58–4.16 (m, 4H), 1.77–1.33 (m, 6H). ^{13}C NMR (75 MHz, $CDCl_3$): δ = 158.1 (2C), 63.3 (2C), 14.1 (2C).

5. References

Analytical data for the synthesized co-crystals and their representative coformers can be found here:

PCA-OXA

- H. Ahmed, M. R. Shimpi, S. P. Velaga. Relationship between mechanical properties and crystal structure in cocrystals and salt of paracetamol. *Drug Dev. Ind. Pharm.* **2017**, *43,* 89.
- A.-S. Persson, H. Ahmed, S. Velaga, G. Alderborn. Powder Compression Properties of Paracetamol, Paracetamol Hydrochloride, and Paracetamol Cocrystals and Coformers. *J. Pharm. Sci.* **2018**, *107,* 1920.
- S. Karki, T. Friščić, L. Fábián, P. R. Laity, G. M. Day, W. Jones. Improving Mechanical Properties of Crystalline Solids by Cocrystal Formation: New Compressible Forms of Paracetamol. *Adv. Mater.* **2009**, *21*, 3905.
- N. Suzuki, M. Kawahata, K. Yamaguchi, T. Suzuki, K. Tomono, T. Fukami. Comparison of the relative stability of pharmaceutical cocrystals consisting of paracetamol and dicarboxylic acids. *Drug Dev. Ind. Pharm.* **2018**, *44*, 582.

PCA-CIT

- S. Schantz, P. Hoppu, A. M. Juppo. A Solid-State NMR Study of Phase Structure, Molecular Interactions, and Mobility in Blends of Citric Acid and Paracetamol. *J. Pharm. Sci.* **2009**, *98*, 1862.
- M. A. Elbagerma, H. G. M. Edwards, T. Munshi, I. J. Scowen. Identification of a new cocrystal of citric acid and paracetamol of pharmaceutical relevance. *CrystEngComm* **2011**, *13*, 1877.

PCA-THP and **PCA-PHE**

- S. Karki, T. Friščić, L. Fábián, P. R. Laity, G. M. Day, W. Jones. Improving Mechanical Properties of Crystalline Solids by Cocrystal Formation: New Compressible Forms of Paracetamol. *Adv. Mater.* **2009**, *21,* 3905.

PXRD-based phase analysis is complemented by literature discussion regarding impurity detection and amorphous content:

- M. Descamps, J. F. Willart, E. Dudognon, V. Caron. Transformation of Pharmaceutical Compounds upon Milling and Comilling: The Role of Tg. *J. Pharm. Sci.* **2007**, *96*, 1398.
- H. G. Brittain. Polymorphism in Pharmaceutical Solids. CRC Press, Boca Raton, **2009**.
- E. Boldyreva, *Chem. Soc. Rev.* **2013**, 42, 7719.
